# A Pilot Clinical Study on the Prognostic Relevance of Plasmatic Exosomes Levels in Oral Squamous Cell Carcinoma Patients

**DOI:** 10.3390/cancers11030429

**Published:** 2019-03-26

**Authors:** Samuel Rodríguez Zorrilla, Mario Pérez-Sayans, Stefano Fais, Mariantonia Logozzi, Mercedes Gallas Torreira, Abel García García

**Affiliations:** 1 Oral Medicine, Oral Surgery and Implantology Unit, School of Medicine and Dentistry, University of Santiago de Compostela, 15782 Galicia, Spain; perezsayans@gmail.com (M.P.-S.); mercedes.gallas.torreira@usc.es (M.G.T.); abel.garcia@usc.es (A.G.G.); 2 Health Research Institute of Santiago de Compostela (IDIS), Santiago de Compostela, 15706 Coruña, Spain; 3 Department of Oncology and Molecular Medicine, Istituto Superiore di Sanitá, 00161 Rome, Italy; stefano.fais@iss.it (S.F.); mariantonia.logozzi@iss.it (M.L.)

**Keywords:** exosomes, oral squamous cell carcinoma, overall survival, OSCC, CD63, CAV-1

## Abstract

Background: To evaluate the relationship between the plasmatic CD63 and CAV1 positive exosome levels, in patients with OSCC before and after surgical treatment and to correlate it with their overall survival. Methods: A double-blind pilot study over 10 patients OSCC and T4 stage without distant metastases or local bone invasion has been performed. The average follow-up period was 37.64 months (34.3–40.84). We obtained 2 plasma tubes of 1 mL each before surgery and 7 days after surgery. Before performing the immunocapture-based analysis, EVs (Extracellular Vesicles) were isolated from the plasma and characterized with western blot analysis. Results: Mean values of CD63 positive plasmatic exosomes (EXO-CD63) after surgery decreased from 750.88 ± 286.67 to 541.71 ± 244.93 (*p* = 0.091). On the other hand, CAV-1 positive plasmatic exosomes (EXO-CAV-1) increased after surgery from 507 ± 483.39 to 1120.25 ± 1151.17 (*p* = 0.237). Patients with EXO-CD63 levels lower than the mean global value before the surgery had a survival of 36.04 months compared with the group with EXO-CD63 higher than the average who only survived 12.49 ± 1.67 months from the diagnosis, *p* = 0.225. When EXO-CAV-1 levels before surgery was lower than the average (813.94 ± 801.21) overall survival was 24.69 ± 22.23 months in contrast when it was higher that was only 11.64 months, *p* = 0.157. Patients with lower EXO-CD63 levels after surgery lived an average of 23.84 ± 23.9 months, while those with higher plasmatic levels of EXO-CD63 live 13.35 months, *p* = 0.808. When EXO-CAV-1 levels after surgery were lower, the average overall survival was 20.344 ± 15.40 months, in contrast when the EXO-CAV-1 levels were higher showing rather an estimate survival expectation of 1.64 months. Conclusions: Surgical treatment induced a dramatic reduction of the plasmatic levels of exosomes expressing CD63 as early as 1 week after resection. This first result suggests that the tumour mass is responsible of the high levels of circulating exosomes detected in cancer patients. At the same time point exosome expressing CAV-1 increased, possibly due to the inflammatory reaction immediately after surgery. Lastly, statistical analysis showed that lower levels of plasmatic exosomes both before and after surgery correlated with a better life expectancy of OSCC patients. Hopefully, this approach will prove useful in the clinical follow-up of cancer patients.

## 1. Background

The most frequent kind of head and neck cancers (HNC) is the squamous cell carcinoma (SCC), it represents 90% of the HNC [1]. Oral cancer is the sixth most common diagnosed cancer in the world and was the cause of 145,000 deaths in 2012, 77% of them happened in regions with a poor economic development [2] According to the US National Cancer Institute, it is estimated that in 2018 there will be a total of 51,540 cases in the country, which represents a total of 3% of new cancer cases. Five-year survival rates for patients with localized oral squamous cell carcinoma are greater than 80% but drop dramatically to 40% when the lymph nodes are involved and to 20% for patients with distant metastasis [2,3,4,5,6].

Exosomes are small nanovesicles from 50–150 nm diameter released into the extracellular microenvironment. They are detectable in body fluids including plasma, saliva, cerebrospinal fluid, ascites but also urine and stools [7,8,9,10]. In fact, many normal cells release exosomes shown to have an active role in regulatory activities of our body; these include dendritic cells, B cells, T cells, mast cells and epithelial cells [11]. However, exosomes are actively released by tumour cells as well [12]. This evidence has suggested that exosomes released by tumour cells may represent a way for cancers to eliminate undesired material or simply their waste [13,14]. Thus, while exosomes definitively have a key role in either cell-to-cell or organ-to-organ communication [8,15], they may have an entirely different role in both cancer progression and metastasis [16,17].

In fact, tumour cells secrete large amounts of exosomes that promote tumour progression [9,18,19,20,21]. Exosomes are an important component of the tumour microenvironment and are one of the main contributors to tumour progression and metastasis [22] delivering an array of molecules potentially involved in cancer pathogenesis [23,24] and able also to transfer genetic material to the germ line [25].

We have set up an immunocapture-based ELISA (Enzyme-Linked Immuno Sorbent Assay) test through which it has been shown that melanoma patients have significantly increased plasmatic levels of exosomes, particularly those expressing a surrogate tumour marker such as CAV-1 (Caveolin-1), as compared with the plasma of healthy donors [26] but also analysing those simply expressing a housekeeping exosomes marker such as CD63 [27].

The discovery up to 10 years ago that exosome contents can be transfers to another cell via fusion to create phenotypic alterations support an intensive research in this field [24,28]. Oral Squamous Cell Carcinoma (OSCC)-derived exosomes are taken up by OSCC cells themselves and significantly promote proliferation, migration and invasion [29]. The exosomes whose content is CAV-1, CD63, Rab5B and Annexin II are the most described in oral cancer research. However, the clinical evidence that exosomes expressing CAV-1 may have a diagnostic potential has not been provided yet, together with the exosome levels during the clinical follow-up before and after surgery.

CAV-1 is expressed in most cell types [30] and is present in a variety of cellular and extracellular compartments. Some studies of CAV-1 in OSCC showed an increased immunoexpression of CAV-1 in OSCC tissue when compared to normal mucosa and precancerous lesions [31] and have shown a gradual increase of expression of CAV-1 in the different steps of cancerous process in oral cancer [32]. In vitro studies have shown that accumulation of CAV1 in tumour microenvironment in in tongue squamous cell cancer had a negative prognostic value [33].

However, while a growing deal of research is involved in the search for new tumour biomarkers, some recent literature suggests that cancer patients have significantly higher plasmatic levels of nanovesicles as compared to controls [27,34] suggesting that the plasmatic levels of exosomes may represent a valuable tumour biomarker [35]. An important example of that is the paper by Osti et al. where is demonstrated that higher extracellular vesicle plasma levels may assist in glioblastoma clinical diagnosis [36]. Notably, the plasmatic levels of tumour exosomes have a direct implication in the metastatic process, both with setting up the so called “metastatic niche” and with the potential of transforming stem cells in the target organs [37,38] 

This study seeks to assess the plasmatic levels of exosomes in patients with oral squamous cell carcinoma. To explore the possibility that the exosome plasmatic levels may be exploited in the follow up of OSCC patients, the same analysis has been performed before and after surgery and related to the patients’ overall survival. To this purpose exosomes were purified from plasma of patients with OSCC before and after surgery and the levels of exosomes quantified using an immunocapture-based test as described [27]. A clinical follow up has been performed in comparing patients with different plasmatic levels of exosomes.

## 2. Methods

### 2.1. Patients

The study consists of 10 patients with oral squamous cell carcinoma with T4 stage without distant metastases or local bone invasion. All of them will undergo tumour resection and lymph node resection surgery if necessary.

Patients diagnosed with OSCC who did not have distant metastases, local bone invasion or any other tumour of any other origin in any part of their body were included in the study from 6 October 2014 to 23 April 2015. Patients with presence of immunological diseases were excluded and patients with treatment history of radiotherapy, chemotherapy or who has used proton pump inhibitors or another antacid drug were excluded too.

### 2.2. Study Protocol

This is a double-blind pilot study to asses de exosomes population before and after surgery and their correlation with the expected patient’s life. The study protocol was approved by the Clinical Research Ethics Committee of Galicia (CAEI) (Ethical code 2018/435). Written informed consent to participate was obtained from all individual participants included in the study. The study has been developed according to the recommendations of STROBE guideline for observational studies.

After developing the clinical history with anamnesis, oral examination, tumour biopsies, radiology techniques and sentinel lymph node of each patient visiting the University Hospital of Santiago de Compostela, the maxillofacial surgeon takes the decision to perform surgery to treat the patient. Before surgery, we obtained 2 plasma tubes of 1 mL each that are kept at −80 degrees Celsius until their transfer to the molecular oncology laboratory of the Instituto Superiore di Sanità in Rome. The same operation is repeated 7 days after the surgery.

### 2.3. Plasmatic Exosomes Characterization and Quantification

Briefly, plasma was centrifuged at 1200× *g* for 20 min followed by 10,000× *g* for 30 min and filtered using a 0.22 mm filter (Millipore Corp., Bedford, MA, USA) and centrifuged at 100,000× *g* for 1 h in a Sorvall WX Ultracentrifuge Series (Thermo Fisher Scientific, Waltham, MA, USA) in order to pellet exosomes. After 1 wash in a large volume of phosphate buffered saline (PBS), exosomes were resuspended in PBS (50–100 mL) or in lysis buffer and stored at 280 uC for experimental analysis. Ninety-six well-plates (Nunc, Milan, Italy) were coated with polyclonal 4 mg/mL anti-Rab-5b antibody (clone A-20, Santa Cruz Biotechnology, Dallas, TX, USA) in a volume of 100 mL/well of carbonate buffer (pH 9.6) and incubated overnight at 4 uC. After 3 washes with PBS, 100 mL/well of blocking solution (PBS containing 0.5% BSA (Bobine Serum Albumin)) were added at room temperature for 1 h. Following 3 washes in PBS, exosomes purified from cell culture supernatants or from plasma were added in a final volume of 50 mL and incubated overnight at 37 uC. After 3 washes with PBS, anti-CD63 Mab (clone H5C6, Pharmingen, San Diego, CA, USA) or anti-caveolin-1 Mab (clone 2297, Pharmingen, San Diego, CA, USA) diluted 4 mg/mL were added and incubated for 1 h at 37 uC. After 3 washes with PBS, the plate was incubated with 100 mL of HRP (Horseradish Peroxidase)-conjugated anti-mouse antibody (Pierce, Milan, Italy) diluted 1:50,000 in blocking solution for 1 h at room temperature. After the final 3 washes with PBS, the reaction was developed with POD (peroxidase) for 15 min (Roche Applied Science, Milan, Italy), blocked with H2SO4 and optical densities (OD) were recorded at 450 nm.

### 2.4. Statistical Analysis

The descriptive statistical analysis was carried out by using the average or median according to the application conditions. We apply the interquartile range and standard deviation in the quantitative variables and frequencies and percentage for the qualitative ones. For the comparison of means we used the t-student test. For the relationship of the expression of markers with the clinical variables, we used the non-parametric test *U* of Mann-Whitney. We carried out a survival study based on Kaplan-Meyer curves. The correlation between the expression of the markers was made by the Pearson test. A logistic regression study was performed using COX regression to evaluate the role of markers expression in global survival. Significant differences were considered with *p* values under 0.05.

## 3. Results

We want to emphasize that the aim of this study was to provide data on the clinical follow up of OSCC based on the characterization and quantification of exosome plasmatic levels before and after surgery. Thus, we first set up the list of patients to be included into the study. The clinical and pathological variables are summarized in Table 1.

**Figure 1:**
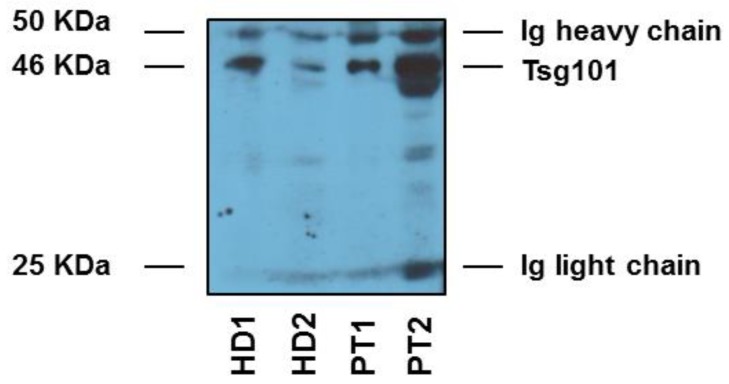
Protein quantification and characterization by Western blot analysis for housekeeping markers of exosomes. Western blot analyses of tumour susceptibility gene 101 (Tsg101). HD1 and 2 are healthy donors; PT 1 and 2 are two patients.

In all patients, exosomes were purified from plasma samples before and after surgery and the plasmatic levels of either CD63 or CAV-1 positive exosomes measured as appropriate, by an immunocapture-based assay. Before performing the immunocapture-based analysis extracellular vesicles were isolated by ultracentrifugation from the plasma of the patients and characterized with western blot analysis of the exosomal markers used in this study (i.e., CD63 and CAV-1), as described [27]. Figure 1 shows a western blot analysis of the expression of a typical exosome marker (i.e., Tsg101) in our plasmatic preparations. As shown in the figure EVs purification from plasma contains both light and heavy Ig chains that entirely cover the expression of both CAV-1 and CD63, that in fact cannot be used in WB analysis of plasmatic EVs. Moreover, CD63 cannot be analysed by WB in as much as the specific antibody needs reducing conditions to work. So, with this analysis we show that our preparations obtained after repeated rounds of ultracentrifugation of patients’ plasma contained exosomes, actually. This, approach was perfectly fitting with the last rules stated in the MISEV position paper [39]. Thus, we carried on the clinical evaluation of the plasmatic exosome levels in patients with OSCC. The results showed that the above markers were clearly detectable in plasmatic exosome preparations.

**Figure 2:**
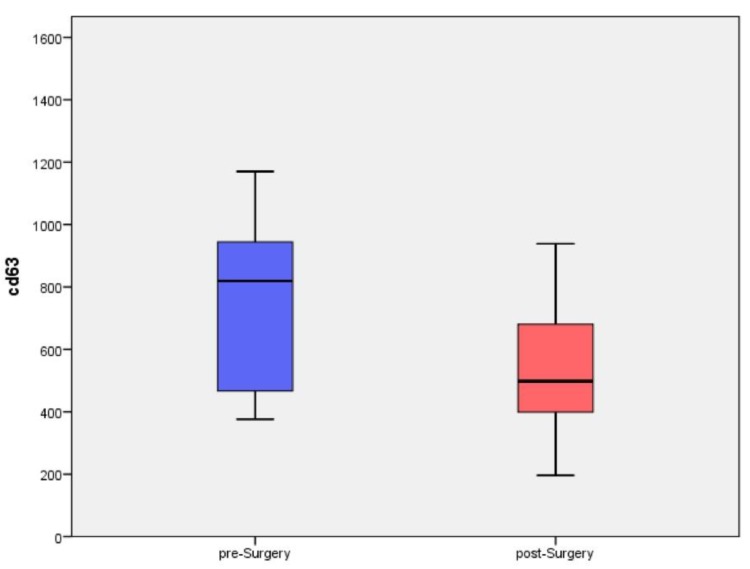
Box plot shows how CD63 positive plasmatic exosomes decrease after surgery from 750.88 ± 286.67 to 541.71 ± 244.93 (*p* = 0.091).

The quantitative evaluation showed that the mean values of CD63 positive plasmatic exosomes after surgery decreased from 750.88 ± 286.67 (median values: 819.5 IQR:437.25–964.25) to 541.71 ± 244.93 (median values: 498 IQR:377–696) (*p* = 0.091), Figure 2. On the other hand, CAV-1 positive plasmatic exosomes increased after surgery from 507 ± 483.39 (median values: 283 IQR:90.5–1023.75) to 1120.25 ± 1151.17 (median values: 494.5 IQR:191.75–1187) (*p* = 0.237).

In terms of correlation, there is a positive correlation between pre-CD-63 and post CD-63 (CC = 0.598, *p* = 0.034). In relation to CAV-1, there is no correlation between pre- and post-surgical levels, although there is a tendency to the inverse correlation, increasing after surgery.

**Figure 3:**
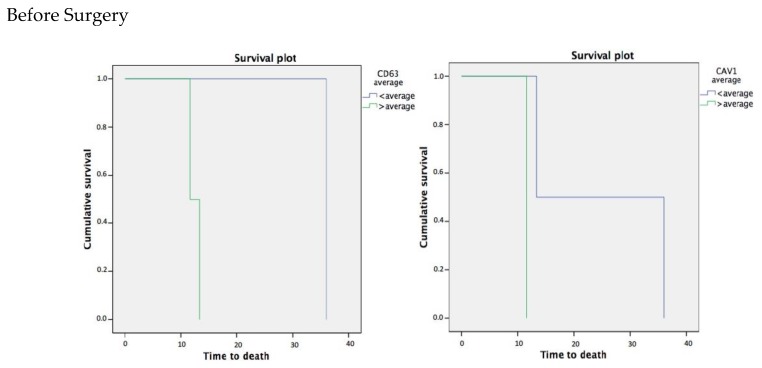
The graph shows the cumulative survival of patients according to their average levels of exosomes with CD-63 (**left**) and CAV-1 (**right**) before resective surgery.

**Figure 4:**
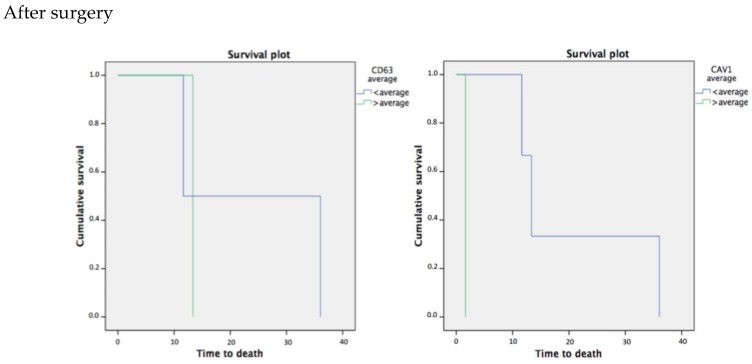
The graph shows the cumulative survival of patients according to their average levels of exosomes with CD-63 (**left**) and CAV-1 (**right**) after resective surgery.

The follow-up period for our patients was from October 2014 to March 2018. The average follow-up time was 37.64 months with a minimum of 34.3 months and a maximum of 40.84 months of follow-up. The standard deviation was 2.67 months. We performed Kaplan Meier Survival Curves to asses difference in survival rates between different exosome plasmatic levels. Results are showed in Figure 3 and Figure 4.

Thus, patients with exosomes CD63+ (CD63+ EXO (Exosome)) amounts lower than the mean global value (653.27 ± 280.123) before the surgery have a HR = 36.041 months, 95% CI (36.041–36.041); *p* = 0.225 which is higher compared to the group with exosomes CD63+ EXO higher than the mean who only survived HR = 12.496 months, 95% CI (10.820–14.172); *p* = 0.225 from the surgical treatment. When the levels of CAV-1+ exosomes (CAV-1+ EXO) before the surgery are lower than the average (813.94 ± 801.21) the expected survival is HR = 24.696 months, 95% CI (2.460–46.932); *p* = 0.157 in contrast when it was higher than 813.94 ± 801.21 that was HR = 11.641 months, 95% CI (11.641–11.641); *p* = 0.157 (Log Rank test significance level = 0.157).

Patients with lower CD63+ EXO levels after surgery have a HR = 23.841 months, 95% CI (0.00–47.753); *p* = 0.808 in comparison with those with high levels of CD63+ EXO with HR = 13.351 months, 95% CI (13.351–13.351); *p* = 0.808. Log Rank test showed a significance of 0.808. Notably, while the CAV-1+ EXO were in average higher than before surgery, the Kaplan-Meier Curve showed that when CAV-1+ EXO levels after surgery were lower than the average the estimate survival was HR = 20.344 months, 95% CI (4.931–35.757); *p* = 0.083 in contrast when the CAV-1+ EXO were higher than the average, with an estimate survival expectation HR = 1.644 months, 95% CI (1.644–1.644); *p* = 0.083, near to the significance level of 0.05.

All in all, while this study was conceived as a pilot clinical study to evaluate the potential use of plasmatic exosome levels in OSCC patients, it provided some important data. First, after the surgical treatment the plasmatic CD63+ EXO levels dropped to lower values, suggesting that the tumour mass was at least in part responsible of the number of circulating exosomes we found before surgery. Then, the highest exosome levels before surgery, either CD63 or CAV-1+ were proven to be related to a poor overall survival. Lastly, after surgery we showed a reduction in CD63+ EXO and an increase in the CAV-1+ EXO, levels but always the exosome plasmatic levels increase after surgery was associated with a clear reduction in the survival.

## 4. Discussion

Our pilot study wanted to analyse the relationships between the plasmatic exosome levels, positive for either CD63 or CAV-1, in patients with stage 4 oral squamous cell carcinoma without distant metastasis or local bone invasion, before and after surgical treatment.

The results showed that the plasmatic levels of CD63+ exosome levels decreased after tumour and lymph node resection surgery, although this decrease does not become significant due to the sample size available. On the other hand, the mean values of plasmatic CAV-1 exosomes increased after surgery approximately. However, the most challenging results came from the relationships between the plasmatic exosome levels before and after surgery and the overall survival of the patients included in this study. In glioblastoma patients supports the role of the plasmatic levels of exosomes rather to the expression of specific markers in the follow up of cancer patients [36]. We need to conduct more studies in order to analyse the relation of this expression with other clinical and pathological markers.

First of all, patients with lower plasmatic levels of both CD63 and CAV-1+ exosomes either before and after surgery showed a longer OS (Overall Survival). This result is supported by previous observation correlating the exosome levels to the disease progression and survival [40,41]. However, these data are also consistent with the evidence that the plasmatic exosome levels are directly related to the tumour mass, as clearly showed in pre-clinical in vivo experiments [27]. The results of this study show both that prognosis is related to the exosome plasmatic levels and that after surgery exosome expressing the ubiquitous CD63 marker decreased after surgical treatment.

The fact that 80% of our patients have significantly increased CAV-1 levels after tumour resection is paradoxical. However, being CAV-1 not a specific exosome marker, as CD63 is actually, it appears conceivable that this paradoxical increase may be due the pro-inflammatory effect of surgical treatment. In fact, CAV-1 has been shown to be upregulated by the hypoxia-inducible factor (HIF)-α [42] that enhances the oncogenic potential of tumour cells by increasing the cell’s proliferative, migratory and invasive capacities [43]. Previous reports support the evidence that in squamous cell carcinoma overexpression of CAV-1 is directly related to the disease progression, in this differing to other tumours [44,45]. Here, in fact, we show that overexpression of CAV-1 on plasmatic exosomes related with a reduced OS. This idea is also supported by the fact that CAV-1 and CD63 data before surgery were highly consistent, also from a prognostic way of thinking. Of course, this hypothesis, while highly conceivable, may only be addressed with a larger number of patients and a different protocol of patients’ follow up with assessment of CAV-1+ exosomes at a various time points after surgical treatments.

Our study adds much to the clinical application of plasmatic exosome measurements in the follow-up of OSCC patients. However, this is probably the first study comparing the plasmatic exosome levels before and after surgery in cancer patients and providing a relationship with the patients’ prognosis. We want to emphasize that this is an entirely non-invasive diagnostic approach that together with offering a potential source for new cancer biomarkers strongly supports a recent hypothesis proposing circulating exosome levels as a valuable tumour biomarker [35]. Recently, the method used for this study (immunocapture-based ELISA) has been compared to other two quantitative methods, such as NTA (Nanoparticle Tracking Analysis) and nanoscale flow cytometry, showing that it is entirely reliable in provide both qualitative and quantitative data in both cell culture supernatants and cancer patients’ plasma [34].

We consider that the analysis of the exosome levels before and after surgery provide valuable clinical information regarding the life expectancy of cancer patients and in particular those suffering OSSC.

With the extension of the study to a higher number of patients and with the implementation of this approach with other methods this non-invasive method will conceivably enter into the future clinical management of cancer patients. Particularly, it will be of paramount importance in the follow up of patients undergoing surgical treatment.

We hypothesized that the exosome levels may represent per se a tumour biomarker [35] and that, on the basis of pre-clinical in vivo evidence [27], the plasmatic exosome levels were related the tumour size [27]. Here we show that the surgical removal of the OSCC tumours drastically reduced the levels of plasmatic exosomes, supporting that preclinical evidence. We proposed to call the amount of plasmatic exosomes in cancer patients, “the circulating tumour mass” [35] and this study provides direct evidence that it might be a denomination respecting a real fact. The huge amount of circulating exosomes in cancer patients may well account for their participation to the metastatic process [17,37]. Considering also that exosomes may deliver genes to the germ line [25]. We want to emphasize as the data of this study show for the first time the exosome plasmatic levels in cancer patients in a longitudinal way, that is, before and after surgery, thus supporting the use of as simple as and as feasible as immunocapture-based ELISA for determining and characterizing exosomes and EVs in general in clinical investigations.

## 5. Conclusions

This study shows that the surgical treatment induced a dramatic reduction of the plasmatic levels of exosomes expressing CD63 as early as 1 week after resection. This first result suggests that the tumour mass is responsible of the high levels of circulating exosomes detected in cancer patients. At the same time point exosome expressing CAV-1 increased, possibly due to the inflammatory reaction immediately after surgery. Lastly, statistical analysis showed that lower levels of plasmatic exosomes both before and after surgery correlated with a better life expectancy of OSCC patients.

Hopefully, this approach will prove useful in the clinical follow-up of cancer patients.

## Figures and Tables

**Table 1:** The table reflects the frequency of the clinical-pathological variables collected from oral squamous cell carcinoma (OSCC) patients.

Variable			Frequency (%)
*N*			10 (100%)
Sex		Male	7 (70%)
		Female	3 (30%)
Age			71.5 (SD = 6.1) 64–84
Smoking habit		Yes	7 (70%)
		No	1 (10%)
		In the past	2 (20%)
TNM	T (size)	1	0 (0%)
		2	0 (0%)
		3	0 (0%)
		4	10 (100%)
	N (lymph nodes)	0	5 (50%)
		1	2 (20%)
		2	1 (10%)
		3	2 (0%)
	M (distant metastasis)	0	10 (100%)
		1	0 (0%)
Location		Mouth floor	3 (30%)
		Upper jaw	3 (30%)
		Palate	1 (10%)
		Jugal mucosa	1 (10%)
		Tongue base	1 (10%)
		Retromolar trigone	1 (10%)
Tumour differentiation		Well differentiated	0 (0%)
		Moderately differentiated	8 (80%)
		Poorly differentiated	2 (20%)

## Abbreviations

BSA	Bobine Serum Albumin
CAV-1	Caveolin-1
ELISA	Enzyme-Linked Immunosorbent Assay
EVs	Extracellular Vesicles
EXO	Exosome
HIF	Hypoxia-Inducible Factors
HNC	Head and Neck Cancers
HRP	Horseradish Peroxidase
NTA	Nanoparticle Tracking Analysis
OS	Overall Survival
OSCC	Oral Squamous Cell Carcinoma
PBS	Phosphate-Buffered Saline
POD	Peroxidase
SCC	Squamous cell Carcinoma
